# Measuring family planning quality and its link with contraceptive use in public facilities in Burkina Faso, Ethiopia, Kenya and Uganda

**DOI:** 10.1093/heapol/czy058

**Published:** 2018-07-13

**Authors:** Timothee Fruhauf, Linnea Zimmerman, Simon Peter Sebina Kibira, Fredrick Makumbi, Peter Gichangi, Solomon Shiferaw, Assefa Seme, Georges Guiella, Amy Tsui

**Affiliations:** 1 Department of Gynecology & Obstetrics, Johns Hopkins School of Medicine, 600 N. Wolfe Street, Baltimore, USA; 2 Department of Population, Family, and Reproductive Health, Johns Hopkins Bloomberg School of Public Health, 615 N Wolfe Street, E4531, Baltimore, USA; 3 Department of Community Health and Behavioural Sciences, School of Public Health, College of Health Sciences, Makerere University, New Mulago Hill Road, Kampala, Uganda; 4 Department of Epidemiology and Biostatistics, School of Public Health, College of Health Sciences, Makerere University, New Mulago Hill Road, Kampala, Uganda; 5 University of Nairobi and Ghent University, Nairobi, Kenya; 6 Department of Reproductive Health and Health Service Management, School of Public Health, Addis Ababa University, Addis Ababa, Ethiopia; 7 Institut Supérieur des Sciences de la Population, Université Ouaga 1 Pr Joseph Ki-Zerbo, Burkina Faso; 8 Department of Population, Family, and Reproductive Health, Johns Hopkins Bloomberg School of Public Health, 615 N Wolfe Street, E4546, Baltimore, USA

**Keywords:** Family planning, contraception, quality, Burkina Faso, Ethiopia, Kenya, Uganda

## Abstract

The individual impacts of several components of family planning service quality on contraceptive use have been studied, but the influence of a composite measure synthesizing these components has not been often investigated. We (1) develop a composite score for family planning service quality based on health facility data from Burkina Faso, Ethiopia, Kenya and Uganda and (2) examine the influence of structural quality on contraceptive practice in these four countries. We used nationally representative cross-sectional survey data of health facilities and women of reproductive age. First, we constructed quality scores for facilities using principal component analysis to integrate 18 variables. Second, we linked women to their closest facility using geo-coordinates. Third, we estimated multivariable logistic regression models to calculate women’s odds ratios for modern contraceptive use adjusting for facilities’ quality and other factors. In Burkina Faso, Ethiopia and Uganda, the odds of using a modern method of contraception was greater if the nearest facility provided high- or medium-quality services compared with low quality in the univariable model. After controlling for possible confounders, the adjusted odds ratios were significant for high quality (aOR: 3.12, *P* value: 0.005) and medium quality (aOR: 2.57, *P* value: 0.009) in Ethiopia and in the hypothesized direction but not statistically significant in Uganda or Burkina Faso, and in the opposite direction in Kenya. A process quality measure—having been visited by a community health worker—was statistically significantly associated with modern contraceptive use in three of the four countries (Burkina Faso aOR: 2.18, *P* value: 0.000; Ethiopia aOR: 1.78, *P* value: 0.000; Uganda aOR: 1.96, *P* value: 0.012). These results suggest that service quality in public facilities may be less relevant to contraceptive use in environments where the universe and reach of providers changes actively. Programs promoting contraception therefore need to consider quality within facility types and their service environments.

**Key Messages**

- Differences in how quality is defined and measured have led to disparate conclusions about the link between family planning service quality and contraceptive use.
- This study constructs a composite measure that synthesizes evidence on the components of family planning service quality and links it with individual level data to explain contraceptive behaviour.
- Unadjusted models show the quality of family planning services in public facilities to be positively associated with the use of modern contraceptive methods and short-acting methods in three of four countries, but once adjusted for covariates, including distance and facility type, quality is positively and significantly associated in Ethiopia only and positively but not significantly in Uganda.
- The quality of family planning services can be an important factor affecting contraceptive use, varying by type of facility and meriting consideration when developing strategies to reduce unmet need for contraception, reach new contraceptive users and sustain current use.

## Introduction

The public health benefits of family planning are well documented; links between birth limiting and spacing and reductions in maternal, neonatal and under-five mortality are clear (Cleland *et al.* 2006; Ahmed *et al.* 2012). Recognizing the importance of promoting such an effective intervention in areas with limited resources, the Family Planning 2020 initiative set the goal of increasing use of modern contraceptives by 120 million additional women in the world’s poorest countries by 2020 (Brown *et al.* 2014).

Although 38.9 million new contraceptive users were added between 2012 and 2016, significant progress remains to be made (Family Planning 2020 2017). Unmet need for contraception is estimated to be as high as 27.7% for married women in sub-Saharan Africa (Bradley *et al.* 2012). Considering the role quality of family planning services in meeting those needs is warranted. Quality arose noticeably at the International Conference on Population and Development in Cairo in 1994 as an important factor affecting contraceptive use (United Nations Population Fund 1994). Since then this association has been extensively examined.

Early on, modelling showed that an increase in the number of available methods would indirectly increase contraceptive prevalence by improving acceptance and continuation of use (Jain 1989). However, later studies linking data on quality of services and individual behaviour reported mixed results. In Peru, Mensch *et al.* (1996) found a weak association between a 3D measure of quality and contraceptive use, but not with an 8D measure of quality. In Morocco, aspects of quality were associated with contraceptive intention, but not contraceptive use (Magnani *et al.* 1999). In Egypt, continuation rates for the pill were related to the number of trained personnel, access to female physicians and the number of methods available (Ali 2001). In contrast, a later study also in Egypt found that a 4D quality index was associated with the use of an intrauterine device (Hong *et al.* 2006). In Tanzania, information provided to clients and technical competence were associated with contraceptive use (Arends-Kuenning and Kessy 2007). Quality of care at initiation of services was linked to continuation of use in the Philippines (RamaRao *et al.* 2003). Finally, a panel study in Bangladesh showed that women who perceived the quality of their care to be higher were more likely to subsequently adopt and continue using a method (Koenig *et al.* 1997). The magnitude of the association between quality of family planning services and contraceptive use is not clear, but many studies indicate that certain elements of quality can influence contraceptive behaviour (RamaRao and Mohanam 2003). Reducing unmet need, reaching new users and maintaining use therefore require some attention to quality.

These differences in findings are largely due to differences in defining and measuring quality. Two frameworks initially laid the foundation for defining quality. In 1966, Donabedian defined quality as resulting from three components: structure (infrastructure and equipment, management, availability of services, counselling), process (interpersonal and technical) and outcome (client satisfaction) (Donabedian 1966). This framework has since been critiqued for excluding some contextual factors tied to healthcare providers and client characteristics (Coyle and Battles 1999). In 1990, Bruce established a new framework specific to family planning. Six factors were identified as critical for patients: choice of method, information given to clients, technical competency of providers, interpersonal relations, follow-up mechanisms and appropriate constellation of services (Bruce 1990). Later in 1993, the International Planned Parenthood Federation added the perspective of providers by establishing the tools needed for providers to provide quality care to their clients (Huezo and Diaz 1993). Since then, suggestions have been made to expand the scope of quality to include other aspects of reproductive health care, the existence of formal standards for quality, the effects of gender relations on care and factors that modify access to services such as distance, provider attitudes and eligibility criteria. While these frameworks have grounded many of the studies on the quality of family planning, their focus differs and no effort has been made to reconcile them. On the contrary, these multiple perspectives have allowed researchers to select individual aspects of quality, often yielding disparate conclusions about the link between quality and contraceptive use (RamaRao and Mohanam 2003; Tumlinson 2016).

**Figure 1.**
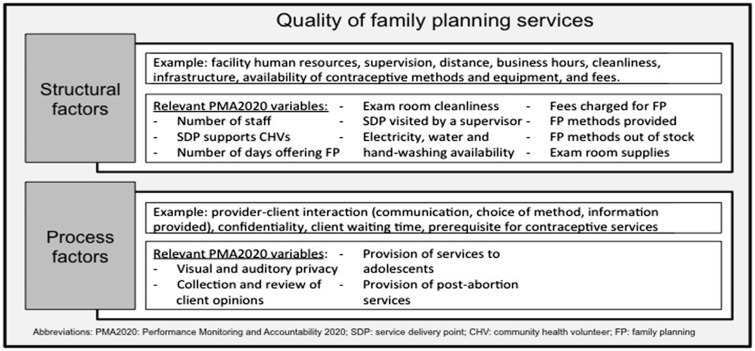
Conceptual framework

A recent literature review on the quality of family planning care in sub-Saharan Africa by Tessema *et al.* (2016) synthesizes the different factors that have been linked to quality, as measured through client satisfaction. Their analysis largely tracks the Donabedian framework and serves as the foundation for the conceptual framework for this study (Figure 1). Factors that affect the quality of family planning can be categorized into three pillars according to this literature review: (1) client, provider and facility characteristics, (2) structural factors and (3) process factors. First, socio-demographic characteristics included age and education status of the client. Second, structural factors included staffing, convenience of available services, cleanliness, infrastructure, contraceptive method-mix and stock, equipment and supplies and fees. Third, process factors included provider–client interactions, confidentiality, client waiting time and eligibility requirements. The review finds client characteristics (Agha and Do 2009; Hutchinson *et al.* 2011; Tafese *et al.* 2013), and the provider’s years of experience (Agha and Do 2009) linked to family planning quality. Similarly, structural factors and facility characteristics such as staff levels, private or public ownership and geographic location, and process factors, such as waiting time, counselling and confidentiality, were tied to quality (Hutchinson *et al.* 2011; Tafese *et al.* 2013; Wang *et al.* 2014).

Despite acceptance of these multifactor frameworks, studies about quality of family planning often select certain aspects of quality or use proxy measures rather than measuring quality as a composite concept. Building on the wealth of data about the variables that affect quality, and in particular the latest synthesis by Tessema *et al.* (2016), this study uses a dimensionality reduction technique to quantify a composite measure of quality on the basis of this evidence. This approach has been used to measure other notions that are difficult to attribute to a single factor (such as household wealth) and appear better suited to reliably assess quality given the multifactorial nature of family planning quality (Creel *et al.* 2002; Vyas and Kumaranayake 2006; RamaRao and Jain 2016; Tumlinson 2016).

Accepted tools to measure family planning quality in resource-poor settings have typically included the Service Provider Assessment Survey (SPA) implemented by ORC Macro International and the Quick Investigation of Quality (QIQ) survey (MEASURE Evaluation 2016). Both include provider interviews, client exit-interviews, provider–client observations and facility audits to obtain a complete overview of family planning. However, these data sets are limited in their ability to examine individual behaviour. Some studies have linked facility data from the SPA and individual level data from the Demographic and Health Surveys (DHS) (Wang *et al.* 2012). However, this is done at the cluster level rather than by individually linking every female respondent to specific facilities due to limited geographic information system (GIS) data; furthermore the SPA and DHS are not typically fielded at the same time additionally limiting inferences. These methodological limitations have previously been identified by researchers and cited as impediments to quantifying the effect of quality on individual contraceptive behaviour (Mensch *et al.* 1996; Hutchinson *et al.* 2011).

This study uses data from Performance Monitoring and Accountability 2020 (PMA2020), a new multi-country platform that surveys female respondents and facilities about family planning with a similar scope to the SPA and QIQ (Zimmerman *et al.* 2017). However, PMA2020 facility and individual level data are collected at the same time and can be linked using GIS data at the individual level. This study is therefore able to analyse associations between the service provision environment and female contraceptive behaviour.

The objective of this study is to determine if the quality of family planning service provision influences the contraceptive behaviour of women in four sub-Saharan countries: Burkina Faso, Ethiopia, Kenya and Uganda. More specifically, it was hypothesized that the higher the quality of family planning provided at the nearest facility, the more likely a woman was to use a modern method of contraception. To examine this link, a composite measure of quality was first created for public facilities offering family planning services and each facility was attributed a quality score. The composite score for each public facility was then linked to sampled female respondents for whom the nearest facility was that public one. The relationship between the quality of that nearest facility and the woman’s modern contraceptive use or her use of short acting methods was then examined.

## Materials and methods

### Data and sampling

Two data sets from PMA2020 were used for each country: a survey of facilities referred to as service delivery points (SDPs) and a survey of women of reproductive age. PMA2020 is a multi-country project that collects a nationally representative sample of data from women aged 15–49 years among selected households and SDPs every six months to one year. Questionnaires are standardized and data are comparable across countries. The female questionnaire collects information on socio-demographic characteristics, reproductive preferences including birth history and contraceptive knowledge, use, history and intention. The SDP questionnaire focuses on facility characteristics, available services, staffing, infrastructure and the provision of family planning services including the availability of contraceptive methods, integration of services and observation of the exam room for family planning visits.

For each country, PMA2020 interviews a probability sample of females 15–49 years and a probability sample of SDPs. A two-stage cluster sampling design is used with enumeration areas (EAs), selected from a frame based on the last census, as the primary sampling unit and households as the secondary sampling unit. Enumeration areas are randomly selected by the country statistical agency within defined strata and all households are listed and mapped. Thirty-five households are randomly selected from the listing—more if the average number of eligible women per household is low—and their occupants enumerated. All women 15–49 years in the selected households are then consented to be surveyed. The four country samples of females for this study are all nationally representative. SDPs are selected on the basis of the probability sample of EAs thereby representing SDPs accessible to the female population in each EA. In all selected EAs, up to three private SDPs are randomly selected and surveyed. In addition, public SDPs for the three lowest levels of care from tertiary to primary SDPs (typically, the health post, intermediate health centre and district or referral hospital) assigned to each selected EA are interviewed. The SDP sample thus is representative of the service environment accessible to the female sample.

This analysis is based on data from four countries’ PMA2020 surveys between late 2015 and mid 2016: Burkina Faso (March–May 2016); Ethiopia (March–April 2016); Kenya (November–December 2015); and Uganda (April–May 2016). The estimation of the composite quality measure was restricted to public SDPs offering family planning services for which information on a set of theoretically relevant factors was available in all four countries. The quality score could not be calculated for private SDPs for which quality-relevant data was not collected. As a result, the influence of the quality of the service provision environment on contraceptive behaviour was only studied among women for whom the nearest SDP (shortest distance between household and facility) was public. The majority of modern contraceptors in these four countries access public facilities for their methods, however: nearly 60% in Uganda, 72.1% in Kenya, 79.5% in Ethiopia and 85.1% in Burkina Faso. More importantly, among women whose closest facility was public, most contraceptors sourced their method from a public facility: 65.5% in Uganda, 74.8% in Kenya, 96.1% in Ethiopia and 86.5% in Burkina Faso. Furthermore, private facilities are geographically concentrated in urban cities and towns. The nearest facility offering family planning services to women is more likely to belong to the public, rather than private, sector (Pomeroy *et al.* 2010; Campbell *et al.* 2015). Public facilities also offer contraceptive methods freely, which in these low-income settings and particularly for rural users, is an attractive option (National Research Council Panel on Reproductive Health 1997). Thus, while facilities in the private sector are important, and increasingly so, the public sector remains a main source for contraceptive information and services. The analysis only included women defined as usual household members and who slept in the house the night before. Table 1 provides the sample sizes. Missing data was highest for the availability of handwashing stations in Burkina Faso (*n* = 9, 8.1%), Ethiopia (*n* = 11, 2.8%) and Kenya (*n* = 6, 2.2%). In Uganda, missing data was highest (*n* = 12, 5.3%) for protection of family planning methods from water, sun and pests.

**Table 1.** Survey dates and sample sizes in the four study countries

	Burkina Faso	Ethiopia	Kenya	Uganda
Survey date	March–May 2016	March–April 2016	November–December 2015	April–May 2016
Number of SDPs	134	461	358	380
Number of public SDPs offering FP services	111	388	267	228
Number of public SDPs offering FP services with complete data	95	371	257	201
Number of women (weighted)	3225 (3237)	7377 (7385)	4845 (4802)	3738 (3745)
Number of women nearest to a public SDP (weighted)^a^	2808 (2988)	6394 (6816)	3509 (3119)	1801 (1810)
Percent of modern users who obtained method from a public SDP	85.1	79.5	72.1	59.6

SDP, service delivery point; FP, family planning.

a The subsample includes women whose nearest SDP was a public one.

### Variables

Eighteen variables from the SDP data set were included to build a composite measure of quality for public SDPs offering family planning services. These variables were selected on the basis of the conceptual framework (Figure 1) informed by research literature. As this study focused on building a measure of service quality using data from SDPs, client characteristics were not included. Similarly, facility characteristics (i.e. public or private ownership and geographic location) were not relevant because the analysis was restricted to public SDPs.

Among the structural factors, the variables included the total number of healthcare staff, whether an SDP supports community health volunteers (CHVs), the number of days per week during which family planning services are offered, two variables assessing cleanliness (presence of clutter or dirt in the examination room and storage of contraceptive supplies away from water, sun, pests and off the floor), whether an SDP was visited by a supervisor in the past six months, whether water and electricity were available on the day of the survey, whether staff had access to handwashing stations, whether fees were charged for family planning services, the total number of contraceptive methods provided, whether an SDP offers long-acting reversible contraceptive (LARC) methods, whether any of the methods provided were out of stock in the past three months, and the total number of supplies observed as available in the examination room. The staffing variable was transformed to the log scale to assure normality and included all healthcare personnel, their titles differing by country. Not all countries were asked about the same contraceptive methods. The list of supplies in the exam room was standardized on the basis of the SPA. Variables representative of process factors included whether an SDP provides visual and auditory privacy in the examination room, has a system to collect, review and report on client opinions, prescribes, counsels on and provides family planning services to unmarried adolescents, and provides post-abortion services.

The primary predictor used to assess the influence of quality on contraceptive use was the quality tertile (low, medium or high) of the nearest public SDP offering family planning for each woman. The main outcome of interest was the woman’s use of a modern method of contraception (mCPR). The use of a short acting modern method of contraception (short-acting mCPR), defined as injectable, pills, emergency contraception, female or male condoms, was a secondary outcome. The relationship between quality and use of short-acting contraceptive methods is hypothesized to be especially important because short-acting methods usually require multiple client–facility interactions making quality of services at a facility a more relevant factor in the decision to use these methods. Secondly, exploring this relationship by analysing quality at the closest facility is specifically appropriate for short-term methods because of higher frequency of interaction, making proximity a relevant factor. Long-acting methods, such as implants, IUDs and sterilization, require usually only one visit and quality of the closest facility may be less relevant. Thirdly, this analysis only includes public facilities which often support CHVs who conduct family planning community outreach and are only authorized to distribute short-term methods such as condoms and pills (Scott *et al.* 2015). This activity is rarely sponsored by private facilities. CHW outreach is captured as a process quality measure as the individual woman’s report of having been visited in the past 12 months and included as a covariate.

Other covariates of interest included distance (kilometres) to the nearest public SDP, which was measured continuously on a logarithmic scale with spline functions. Spline terms were used to approximate linearity because log distance did not appear to have a single linear relationship with mCPR. Breakpoints in the spline functions were selected by examining the LOWESS curve of mCPR by log distance and varied by country. Facility type for the nearest public SDP offering family planning was another covariate and was categorized by level of care (from level 1 to 4, with the first level being akin to a district or referral hospital and the lowest being a health post or clinic) and differed by country. Additional individual-level variables served as controls: age, marital status, highest level of education, urban or rural place of residence and household wealth quintile for each woman.

### Analyses

The composite quality measure was constructed by country using principal component analysis (PCA) including 18 variables selected on the basis of the conceptual framework. These variables were normalized and coded with similar valence (e.g. larger values for continuous variables and higher categories for categorical variables were associated with higher quality). The same variables were included for all countries unless the SD was null. The factor loadings for the principal component that explained the highest proportion of the variance in each SDP sample were used as weights to calculate a normalized SDP quality score. The quality score was categorized by tertile and each public SDP classified as low, medium and high quality by country. Cronbach’s alpha was calculated as a measure of consistency for the variables included in the factor analysis.

To study the influence of quality on contraceptive use, we matched women whose nearest SDP was public to that facility and treated the latter facility’s characteristics as representing the woman’s service environment. The geo-coordinates of the household and SDP were used to calculate the minimum straight-line distance to determine nearest. This approach assumes that the closest service provision environment captures the health system’s strength in contraceptive access and can affect women’s’ contraceptive behaviours. Univariable and multivariable logistic regression models are used to calculate the odds ratios (ORs) and adjusted odds ratio (aORs) of using a modern method of contraception or a short-acting modern method of contraception. Variance inflation factors (VIF) were calculated for all covariates to assess multicollinearity. *P* values with alpha of 0.05 and 0.01 are reported. Weighted results are reported to account for the stratified two-stage cluster sampling design and the variances of the covariates are adjusted accordingly. Missing values were handled using listwise deletion. Data analyses were performed with Stata 12.1 (College Station, TX: StataCorp LP).

## Results

### A composite measure of quality

The characteristics of the samples of public SDPs offering family planning services are presented in Table 2 for the four study countries. On an average, 111 SDPs in Burkina Faso offered 7.6 methods (SD: 1.3) on 6.8 days/week (SD: 0.5) and had a total log staff size of 3.6 (SD: 1.4). SDPs varied most with respect to support to CHVs (*n* = 46, 42.2%), exam room cleanliness (*n* = 70, 64.2%), water availability (*n* = 70, 63.1%), absence of stock outs for all methods provided (*n* = 77, 69.4%) and availability of family planning services for unmarried adolescents (*n* = 72, 64.9%). On an average in Ethiopia, 388 SDPs offered 6.5 methods (SD: 1.6) on 5.4 days/week (SD: 1.0) and had a total log staff of 2.9 (SD: 1.6). The most variation was recorded for support to CHVs (*n* = 109, 28.1%), exam room cleanliness (*n* = 53, 13.7%), availability of electricity (*n* = 210, 54.1%), water (*n* = 194, 50.0%) and handwashing stations (*n* = 297 78.8%), absence of stock outs (*n* = 217, 55.9%), service provision to unmarried adolescents (*n* = 175, 45.1%) and post-abortion (*n* = 285, 73.5%). On an average, 267 SDPs in Kenya offered 6.9 methods (SD: 1.5) on 5.2 days/week (SD: 0.6) and had a total log staff size of 2.2 (SD: 1.3). SDPs varied most in their support of CHVs (*n* = 137, 51.3%), water availability (*n* = 186, 69.7%), absence of stock outs (*n* = 146, 54.7%) and provision of services to unmarried adolescents (*n* = 152, 56.9%) and post-abortion (*n* = 176, 65.9%). On an average in Uganda, 228 SDPs offered 5.6 methods (SD: 2.3) on 5.5 days/week (SD: 1.2) and had a total log staff of 2.7 (SD: 1.1). Most variation was recorded for support to CHVs (*n* = 134, 58.8%), electricity (*n* = 140, 61.4%) and water (*n* = 103, 45.2%) availability, provision of LARC methods (*n* = 150, 65.8%), absence of stock outs (*n* = 54, 23.7%) and provision of services post-abortion (*n* = 158, 69.3%) and to unmarried adolescents (*n* = 161, 70.6%).

**Table 2.** Sample characteristics of public service delivery points offering family planning in the four study countries

	Burkina Faso	Ethiopia	Kenya	Uganda
Number of public SDPs offering FP, *N*	111	388	267	228
Number of staff, log mean (SD)	3.6 (1.4)	2.9 (1.6)	2.2 (1.3)	2.7 (1.1)
SDP supports CHVs, *n* (%)	46 (42.2)	109 (28.1)	137 (51.3)	134 (58.8)
Weekly number of days offering FP mean (SD)	6.8 (0.5)	5.4 (1.0)	5.2 (0.6)	5.5 (1.2)
FP exam room is not cluttered or dirty, *n* (%)	70 (64.2)	53 (13.7)	243 (91.0)	178 (80.9)
FP methods are protected from water, sun, pests and off the floor, *n* (%)	93 (87.7)	313 (81.5)	209 (78.9)	186 (86.1)
SDP visited by a supervisor, *n* (%)	109 (98.2)	366 (94.3)	251 (94.7)	220 (97.8)
Electricity is available, *n* (%)	92 (82.9)	210 (54.1)	224 (83.9)	140 (61.4)
Water is available, *n* (%)	70 (63.1)	194 (50.0)	186 (69.7)	103 (45.2)
Handwashing station is available for staff, *n* (%)	101 (99.0)	297 (78.8)	259 (99.2)	217 (98.2)
No fees for FP, *n* (%)	65 (58.6)	384 (99.0)	247 (92.5)	227 (99.6)
Number of FP methods provided mean (SD)	7.6 (1.3)	6.5 (1.6)	6.9 (1.5)	5.6 (2.3)
LARC offered, *n* (%)	111 (100.0)	364 (93.8)	261 (97.8)	150 (65.8)
No FP method out of stock, *n* (%)	77 (69.4)	217 (55.9)	146 (54.7)	54 (23.7)
Number of supplies in FP exam room mean (SD)	10.5 (1.6)	8.4 (2.1)	9.8 (1.4)	8.1 (1.7)
Visual and auditory privacy available, *n* (%)	109 (100.0)	343 (88.4)	260 (97.4)	198 (90.0)
Adolescents are prescribed, counselled on, provided FP, *n* (%)	72 (64.9)	175 (45.1)	152 (56.9)	161 (70.6)
Post-abortion services available, *n* (%)	107 (97.3)	285 (73.5)	176 (65.9)	158 (69.3)
SDP collects, reviews and reports on client opinions, *n* (%)	67 (60.4)	228 (58.8)	139 (52.1)	94 (41.4)

SDP, service delivery point; FP, family planning; SD, standard deviation; CHV, community health volunteer.

**Figure 2.**
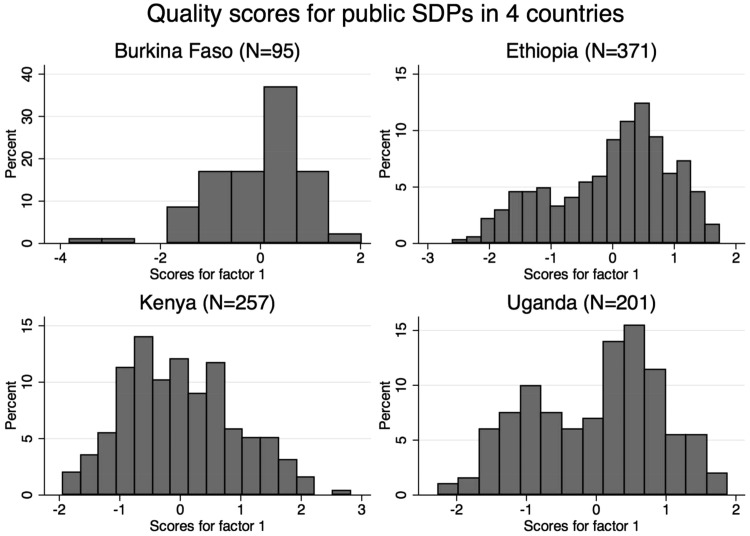
Quality scores for public SDPs in four countries

Using PCA, we identified the first component explaining the most variance among SDPs for the 18 included variables by country (Table 3). The first component explained 78.9% of the variance in Ethiopia, 63.4% in Uganda, 45.7% in Kenya and 42.1% in Burkina Faso. Cronbach’s alpha values ranged from 0.60 (Burkina Faso) to 0.75 (Ethiopia). Factor loadings differed by country, but there were common trends. Staffing was the highest or second highest loading variable in all countries (0.7546–0.8713). The total number of contraceptive methods (0.4929–0.8535), the availability of water (0.4504–0.6784) or electricity (0.3516–0.6339), and the provision of post-abortion services (0.4594–0.6354) also consistently loaded highly across the four countries. In Ethiopia the provision of family planning services free-of-charge, in Kenya the number of supplies in the exam room and in Uganda the provision of LARCs also had high loadings. For each SDP, a normalized quality score was calculated on the basis of the loadings for the first principal component by country. The distribution and estimates by tertile are presented in Figure 2.

**Table 3.** Principal component analysis for the quality score for public service delivery points offering family planning in the four study countries

	Burkina Faso		Ethiopia	Kenya		Uganda
Principal component^a^	PC1	PC2	PC1	PC1	PC2	PC1
Number of public SDPs offering FP, *N*	95		371	257		201
Eigenvalue	2.22	1.31	4.07	2.67	2.14	2.85
Proportion of variance (%)	42.1	24.8	78.9	45.7	36.7	63.4
Factor loadings						
Number of staff, log	0.7862	−0.2880	0.8713	0.7546	0.2753	0.7580
SDP supports CHVs	−0.1594	0.3652	−0.1831	−0.0071	0.5469	0.1105
Weekly number of days offering FP	−0.2570	−0.3273	−0.4028	−0.3568	−0.0922	0.1348
FP exam room is not cluttered or dirty	0.1546	−0.0667	0.3841	0.4059	−0.5684	0.1641
FP methods are protected from water, sun, pests and off the floor	−0.2448	0.0082	0.0664	0.0723	0.1350	−0.0479
SDP visited by a supervisor	0.0605	−0.0360	−0.0058	0.1590	0.3473	0.0609
Electricity is available	0.3992	−0.3266	0.6339	0.4799	−0.1620	0.3516
Water is available	0.6784	−0.2159	0.6030	0.5554	−0.3672	0.4504
Handwashing station is available for staff	0.3949	0.6025	0.6051	−0.0596	−0.0124	0.1279
No fees for FP	0.0034	−0.1425	−0.0456	−0.3348	−0.0145	−0.0364
Total number of FP methods provided	0.5170	−0.2197	0.8180	0.4929	0.6201	0.8535
LARC offered	–		0.3806	0.0720	0.2763	0.7566
No FP method out of stock	−0.1356	0.0326	0.1380	0.2502	−0.2962	−0.2021
Number of supplies in FP exam room	0.2203	0.1847	0.5758	0.5381	−0.2597	0.4276
Visual and auditory privacy available	–		0.2177	0.0967	−0.0611	0.2060
Adolescents are prescribed, counselled on, provided FP	0.2718	0.2239	−0.0461	0.3869	−0.5152	0.0168
Post-abortion services available	0.4721	0.5035	0.6354	0.4594	0.3640	0.4799
SDP collects, reviews and reports on client opinions	0.1371	0.2415	0.4647	0.3867	0.3399	0.2722
Cronbach’s alpha	0.5967		0.7490	0.6556		0.6470

PC, principal component; SDP, service delivery point; CHV, community health volunteer; FP, family planning.

– represents no variation in variable (i.e. 100% of SDPs in one category), variable was dropped from the PCA.

a Only the components with Eigen value > 1 are showed.

### The influence of quality on contraceptive use

The characteristics of women 15–49 years whose nearest SDP was public are reported in Table 4. In Burkina Faso, 20.8% of women 15 to 49 years reported using a modern method of contraception (the modern contraceptive prevalence rate or mCPR) and 11.6% a short acting method. For almost two-thirds of women (*n* = 1897, 66.8%) their nearest SDP scored low on quality, with 624 (22.0%) women close to a medium-quality SDP, and 317 (11.2%) close to a high-quality SDP. Most women lived nearest to health and social promotion centres (*n* = 2529, 84.6%) followed by medical centres (*n* = 405, 13.6%). In Ethiopia, the mCPR was 26.1% and short-acting CPR was 20.1%. For more than the majority of women (*n* = 3892, 58.6%) the nearest public SDP provided family planning services of low quality; 1863 (28.1%) and 887 (13.4%) women lived nearest to an SDP providing family planning services of medium and low quality, respectively. Approximately half lived closest to a health post (*n* = 3442, 50.5%) and 3082 (45.2%) to a health centre. In Kenya, 44.8% of women reported using a modern method and 30.7% were using a short acting method. The quality of the nearest public SDP was more equitably distributed with 1071 (35.0%), 1173 (38.3%) and 821 (26.8%) women living close to an SDP providing low, medium- and high-quality family planning services, respectively. The closet public SDP was a pharmacy for the majority of women (*n* = 1757, 56.3%), followed by a health centre (*n* = 932, 29.9%) and a hospital (*n* = 391, 12.5%). In Uganda, the mCPR was 24.2% and short-acting CPR was 19.3% among women 15–49 years. Slightly less than half (*n* = 798, 48.3%) of women lived near a low-quality SDP, 670 (40.6%) closest to a medium-quality SDP, and 183 (11.1%) closest to an SDP providing high-quality family planning services. Most women (*n* = 769, 42.5%) lived closest to the lowest level (level two) health centre, followed by 667 (36.8%) for level three health centres and 269 (14.8%) for level four health centres. Only 106 (5.8%) women had their closest SDP being a district hospital. The percent of women reporting being visited by a community health worker in the past year ranged from 10.7 in Kenya to 18.2 in Ethiopia.

**Table 4.** Sample characteristics of women 15–49 years whose nearest service delivery point offering family planning is public in the four study countries

	Burkina Faso	Ethiopia	Kenya	Uganda
Number of women 15–49 years, *N*	2988	6816	3119	1810
mCPR, *n* (%)	623 (20.8)	1782 (26.1)	1396 (44.8)	439 (24.2)
Short-acting mCPR, *n* (%)	314 (11.6)	1283 (20.1)	914 (30.7)	310 (19.3)
Quality tertile of nearest SDP, *n* (%)				
Low	1897 (66.8)	3892 (58.6)	1071 (35.0)	798 (48.3)
Medium	624 (22.0)	1863 (28.1)	1173 (38.3)	670 (40.6)
High	317 (11.2)	887 (13.4)	821 (26.8)	183 (11.1)
Distance to SDP, log km^a^ mean (SD)	0.87 (0.15)	0.28 (0.07)	0.35 (0.08)	0.83 (0.15)
	0.10 (0.05)	0.31 (0.04)	0.03 (0.02)	0.08 (0.03)
Type of nearest SDP, *n* (%)				
Level 1	39 (1.3)	292 (4.3)	391 (12.5)	106 (5.8)
Level 2	405 (13.6)	3082 (45.2)	932 (29.9)	269 (14.8)
Level 3	2529 (84.6)	3442 (50.5)	39 (1.3)	667 (36.8)
Level 4	15 (0.5)	–	1757 (56.3)	769 (42.5)
Visited by a community health worker, *n* (%)	466 (15.6)	1240 (18.2)	335 (10.7)	323 (17.9)
Age mean (SD)	28.0 (0.26)	28.0 (0.17)	28.2 (0.19)	28.2 (0.31)
Marital status, *n* (%)				
Currently married/living with partner	2320 (77.7)	4441 (65.2)	1831 (58.7)	1257 (69.4)
Divorced/Separated/Widowed	77 (2.6)	628 (9.2)	224 (7.2)	162 (9.0)
Never married	591 (19.8)	1748 (25.6)	1065 (34.1)	391 (21.6)
Highest level of school attended, *n* (%)				
Never attended/preschool	1955 (65.5)	2999 (44.0)	128 (4.1)	216 (12.0)
Primary	518 (17.4)	2625 (38.5)	1661 (53.3)	1189 (65.8)
Secondary	487 (16.3)	965 (14.2)	896 (28.7)	358 (19.8)
Technical/vocational	–	144 (2.1)	66 (2.1)	32 (1.8)
Higher	24 (0.8)	79 (1.2)	368 (11.8)	11 (0.6)
Place of residence, *n* (%)				
Urban	546 (18.3)	1249 (18.3)	847 (27.1)	128 (7.1)
Rural	2442 (81.7)	5568 (81.7)	2272 (72.9)	1682 (92.9)
Household wealth quintile/tertile, *n* (%)				
Poorest	1137 (38.0)	1425 (20.9)	851 (27.3)	456 (25.2)
Poorer	–	1419 (20.8)	796 (25.5)	401 (22.2)
Middle	974 (32.6)	1402 (20.6)	702 (22.5)	436 (24.1)
Richer	–	1352 (19.8)	403 (12.9)	295 (16.3)
Richest	877 (29.3)	1218 (17.9)	369 (11.8)	221 (12.2)

mCPR, modern contraceptive prevalence rate; SDP, service delivery point; FP, family planning.

– represents category not defined in this country.

a Two spline terms were defined by country to approximate linearity

Facility types by country: Burkina Faso: 1) Hospital and polyclinic, 2) Medical centre, 3) Health and social centre, 4) Pharmacy; Ethiopia: 1) Hospital and polyclinic, 2) Health centre, 3) Health post; Kenya: 1) Hospital, 2) Health centre, 3) Health clinic, 4) Pharmacy; Uganda: 1) Hospital, 2) Health centre 4, 3) Health centre 3, 4) Health centre 2.

The quality of family planning services in public SDPs was positively associated with modern contraceptive use in Burkina Faso, Ethiopia and Uganda in the univariable analysis (Table 5). In Burkina Faso the odds for women using modern methods of contraception was significantly higher if they lived closest to a public SDP providing high-quality family planning services than one providing low-quality services (OR = 1.91, *P* < 0.000). In Ethiopia, the odds of using modern contraception were significantly greater among women who lived closest to a medium-quality SDP compared with women who lived closest to a low-quality SDP (OR = 1.39, *P* = 0.037). After adjustment for control covariates, the relationship strengthened and remained statistically significant in Ethiopia (Table 5). The odds of using modern contraception were 2.57 (*P* = 0.009) times higher among women living closest to an SDP providing medium-quality services compared with women living closest to an SDP providing low-quality services. The odds of using contraception were even higher for women living closest to a high-quality SDP than a low-quality SDP (aOR = 3.12, *P* = 0.005).

**Table 5.** Unadjusted and adjusted^a^^,b^ odds ratio from logistic regression of modern contraceptive use on selected covariates in four study countries

	Burkina Faso		Ethiopia		Kenya		Uganda	
	OR	*P* value	OR	*P* value	OR	*P* value	OR	*P* value
Number of women 15–49 years, *N*	2542		6179		3375		1652	
Quality tertile of nearest SDP (ref: low)								
Medium	1.47	0.108	1.39**	0.037	0.70**	0.043	0.88	0.590
High	1.91***	0.000	1.27	0.147	0.81	0.275	1.15	0.644

	aOR	*P* value	aOR	*P* value	aOR	*P* value	aOR	*P* value

Number of women 15–49 years, *N*	2540		6173		3375		1650	
Quality tertile of nearest SDP (ref: low)								
Medium	0.697	0.22168	2.57***	0.009	0.76	0.1546	0.971	0.845637
High	0.612	0.1476	3.120***	0.005	0.53***	0.0086	1.1405	0.727889
Visited by a community health worker (ref: no)	1.71***	0.003	1.72***	0.000	1.02	0.918	1.99**	0.018
Distance to nearest SDP, spline 1, log km	0.720***	0.0053	1.089	0.404358	0.89**	0.045	1.06	0.63761
Distance to nearest SDP, spline 2, log km	1.8095	0.17869	0.64	0.132085	3.065***	0.000	0.820	0.803772
Type of nearest SDP (ref: level 1)^c^								
Level 2	1.004	0.986870	1.540	0.33271	0.86	0.57061	10.0284	0.9758
Level 3	0.435***	0.001	32.1894**	0.0215	1.243	0.41826	10.0284	0.97740
Level 4	–	–	–	–	0.754	0.28062	10.1893	0.78590

SDP, service delivery point; FP, family planning; OR, odds ratio; aOR, adjusted odds ratio.

a Adjusted for age, marital status, highest level of education, place of residence, household wealth quintile.

b Variance inflation factors for all included covariates were <10 indicating absence of multicollinearity.

c Type of nearest SDP by country: Burkina Faso: 1) Hospital and polyclinic, 2) Medical centre, 3) Health and social centre, 4) Pharmacy; Ethiopia: 1) Hospital and polyclinic, 2) Health centre, 3) Health post; Kenya: 1) Hospital, 2) Health centre, 3) Health clinic, 4) Pharmacy; Uganda: 1) Hospital, 2) Health centre 2, 3) Health centre 3, 4) Health centre 4.

– represents category not defined in this country.

*** *P*<0.01,

** *P*<0.05.

The relationship was reversed in Kenya: the adjusted odds of contraceptive use were unexpectedly lower among women who lived closest to a facility providing high-quality family planning services compared with women living closest to a low-quality facility (aOR = 0.53, *P* = 0.008).

Being visited by a community health worker in the past year was significantly and positively associated with the odds of using modern contraception in three countries—Burkina Faso (aOR = 1.71, *P* = 0.003), Ethiopia (aOR = 1.72, *P* = 0.000) and Uganda (aOR = 1.99, *P* = 0.018)—suggesting outreach serves as an important predictor of process quality.

Distance to the nearest SDP measured with spline functions and two spline breakpoints captured significant change in the slopes of the SDP’s distance in relation to use in Burkina Faso and Kenya but not Ethiopia and Uganda. The second spline breakpoint in Burkina Faso and Kenya had higher aORs than the first suggesting that women living far from an SDP in these countries may be making greater effort to use contraception.

The main contraceptive methods used in all four countries were implants, injectables, pills and male condoms, with injectables dominating the method mix in all countries except Burkina Faso where implants were the most used method (PMA2020 2017). As visible from Table 4, short-acting method use comprises the majority of contraceptive prevalence for this sample as well. When restricting the outcome to short-acting modern contraceptives, quality was positively associated with contraceptive use in Burkina Faso, Ethiopia and Uganda and negatively associated in Kenya in the univariable analysis (Table 6). This relationship remained significant in the multivariate analysis in Ethiopia: women who live closest to a medium-quality SDP or a high-quality SDP had 2.48 or 2.94 greater odds of using a short-acting method of contraception, respectively, than women living closest to an SDP providing low-quality family planning services (aOR = 2.48, *P* = 0.003; aOR = 2.94, *P* = 0.006). In Burkina Faso, women who lived closest to a medium-quality SDP had lower odds of using short-acting contraceptives compared with women living nearest to an SDP providing low-quality services (aOR = 0.52, *P* = 0.019). Similarly, in Kenya, the odds of using a short-acting contraceptive were lower among women living closest to a high-quality SDP compared with a low-quality SDP after adjusting for covariates (aOR = 0.63, *P* = 0.048). Sensitivity analyses including all nearest SDPs regardless of their managing authority (public vs private) revealed no change in regression modelling for modern contraceptive use or short-acting contraceptive use. Similarly, restricting the models to include only contraceptive users showed no change (results not shown).

**Table 6.** Unadjusted and adjusted^a^^,b^ odds ratio from logistic regression of short acting modern method use on selected covariates in four study countries

	Burkina Faso		Ethiopia		Kenya		Uganda	
	OR	*P* value	OR	*P* value	OR	*P* value	OR	*P* value
Number of women 15–49 years, *N*	2305		5823		3227		1481	
Quality tertile of nearest SDP (ref: low)								
Medium	1.04	0.880	1.32	0.094	0.74	0.057	1.00	0.996
High	1.88***	0.002	1.20	0.296	0.94	0.719	1.59	0.100

	aOR	*P* value	aOR	*P* value	aOR	*P* value	aOR	*P* value

Number of women 15–49 years, *N*	2303		5820		3227		1479	
Quality tertile of nearest SDP (ref: low)								
Medium	0.520***	0.0109	2.4850***	0.003	0.84	0.24954	1.1408	0.550767
High	0.678	0.42381	2.945***	0.0065	0.63**	0.0487	1.3625	0.412557
Visited by a community health worker (ref: no)	2.18***	0.000	1.78***	0.000	0.96	0.786	1.96**	0.012
Distance to nearest SDP, spline 1, log km	0.730**	0.03019	1.07	0.506455	0.90	0.271	1.07	0.52037
Distance to nearest SDP, spline 2, log km	1.863	0.37622	0.6258	0.114068	4.168***	0.000	0.643	0.4403
Type of nearest SDP (ref: level 1)^c^								
Level 2	0.7682	0.24871	1.3725	0.358477	0.97	0.9015	0.8978	0.794527
Level 3	0.504***	0.00143	2.9469**	0.0138	0.745	0.3268	0.766	0.588358
Level 4	–	–	–	–	0.867	0.62231	10.0182	0.984668

SDP, service delivery point; FP, family planning; OR, odds ratio; aOR, adjusted odds ratio.

a Adjusted for age, marital status, highest level of education, place of residence, household wealth quintile.

b Variance inflation factors for all included covariates were <10 indicating absence of multicollinearity.

c Type of nearest SDP by country: Burkina Faso: 1) Hospital and polyclinic, 2) Medical centre, 3) Health and social centre, 4) Pharmacy; Ethiopia: 1) Hospital and polyclinic, 2) Health centre, 3) Health post; Kenya: 1) Hospital, 2) Health centre, 3) Health clinic, 4) Pharmacy; Uganda: 1) Hospital, 2) Health centre 2, 3) Health centre 3, 4) Health centre 4.

– represents category not defined in this country.

*** *P*<0.01,

** *P*<0.05.

Service quality at the nearest SDP tended to show the same associations for women’s use of modern contraception as with their use of a short-acting method. There was a slightly stronger relationship in Burkina Faso where the aOR associated with medium, compared with low, quality declined in magnitude from 0.69 to 0.52 and became statistically significant (*P* = 0.019). The spline terms for distance had similar aOR values for the short-acting method use model as they did for the overall modern method use model.

As with any modern method of contraception, women who were visited by a CHW in the past 12 months were significantly more likely to use short-acting modern method of contraception in Burkina Faso (aOR = 2.18, *P* = 0.000), Ethiopia (aOR = 1.78, *P* = 0.000) and Uganda (aOR = 1.96, *P* = 0.012) but not in Kenya.

Because of the relatively lower variance explained by the first component of the PCA for Burkina Faso and Kenya, we also constructed a second quality score for these countries based the second component (Table 3). This component explained another 24.8% of the variance in Burkina Faso and 36.7% in Kenya. However, high loadings were limited to two items in Burkina Faso—whether an SDP offers post-abortion services and handwashing stations—and three items in Kenya—the number of contraceptive methods provided, the cleanliness of the exam room, and whether an SDP supports CHVs. Once divided into tertiles and included in the multivariate analysis, quality as measured by the second component was not significantly associated with either outcomes (results not shown).

## Discussion

This study first attempts to quantitatively measure quality of family planning services in four countries by synthesizing multiple factors identified in the literature into a composite index using PCA. The quality score constructed explains a large proportion of the variance across the four SDP samples. Whether with one or two factors, the proportion of variance explained ranged from 63.4% to 82.4% and Cronbach’s alpha values of internal consistency were high. Second, the study examines the relationship between service quality and contraceptive behaviour by linking health facilities and women. Most importantly, this study shows that is possible to create an index of quality with the same underlying variables to facilitate comparisons and assess patterns of associations with individual level contraceptive outcomes across different settings.

The need for a composite measure of quality is evidenced by the multiple elements that are typically listed in accepted frameworks on family planning quality. These frameworks adopt different perspectives that need to be integrated to achieve a complete understanding of quality. Much attention has been paid to the six components of quality detailed by the Bruce framework (Bruce 1990) from the client’s perspective and the three categories characterized by the Donabedian framework (Donabedian 1966) that mix provider and client approaches. However, many researchers have pointed out elements excluded by these frameworks or conduct studies focused on just one element. On the basis of the components identified in the latest systematic review by Tessema *et al.* (2016), this study creates a quality score able to integrate across multiple perspectives and approaches.

Notably, many of the variables that were given the highest loading or weight across countries on the basis of the first principal component were structural in nature (staffing, family planning methods and functionality represented by water and electricity availability) and elements tied to process had lower weights with the exception of provision of post-abortion services. Several studies have emphasized the breadth of methods available as one of the most important aspects of quality suggesting that facilities offering more methods may be of higher quality (Magnani *et al.* 1999; Ali 2001; Blanc *et al.* 2002; Mugisha and Reynolds 2008; Tafese *et al.* 2013; RamaRao and Jain 2016). Indeed, studies often reach conclusions on service quality on the basis of the method mix offered by facilities (Steele *et al.* 1999; Ali 2001; Hancock *et al.* 2015). The number of methods available was found in this study to have a consistently high loading across the four countries, empirically underscoring the importance of offering multiple methods, especially as this relates to supporting continued use of contraception. However, there remains a need to measure quality from multiple angles, particularly from clients, and the findings from this analysis establish a place for a composite index of service quality.

Moreover, this analysis overcomes the empirical constraint of maximum variation in individual behaviour that can be explained when quality is measured at the cluster level by linking every woman individually to her closest public service provision environment. In linking each woman to her nearest facility, this approach also avoids restricting the analysis to the behaviour of contraceptive users, a constraint that affects studies measuring both quality and contraceptive use through client samples. Finally, this analysis also overcomes some of the selection biases that affect studies that rely on interviews of facility users to measure the quality of those same facilities.

This study found a positive association between the quality of the public family planning service provision environment and contraceptive use in Burkina Faso and Ethiopia at the univariable level. However, after adjusting for distance, facility type and other common covariates at the individual- and facility-level, service quality was only significantly and positively associated with a woman’s odds of contraceptive use in Ethiopia while positively but not with statistical significance in Uganda. While prior studies have addressed quality and contraceptive use, direct comparison of their findings to this analysis is difficult due to methodological differences in measuring quality and the multilevel nature of this study’s data. Nevertheless, the regression analysis provides additional evidence regarding the importance of the quality of services for overall contraceptive use and short-acting method use, in the same direction as the most recent studies linking facility and individual data on intrauterine device use in Egypt (Hong *et al.* 2006), modern contraceptive use in Tanzania (Arends-Kuenning and Kessy 2007), continuation of pills or injectables in Nepal (Gubhaju 2009), and uptake of modern contraception at follow up in the Philippines (RamaRao *et al.* 2003).

The study found that quality of family planning services in public facilities was inversely related to contraceptive use in Kenya. This unexpected observation most likely reflects the growing importance of LARCs, particularly implants, and the exclusion of private sector SDPs from the analysis. Indeed, in the core PMA2020 survey of women of reproductive age, 35% of them were found to purchase their method of contraception from a private facility and among women living closest to a public facility, 25% obtained their method from a private source. As a result, analysing the public service provision environment in isolation provides a necessary but partial perspective on understanding the contraceptive behaviour of women in Kenya. The growth of the Kenyan private sector and the development of informal provider or social marketing systems that often deliver implants and injectables (Agha and Do 2009) has resulted in an increase in provider density that may require reconsidering the assumption that the service provision environment affecting use is defined by the nearest facility, even if private facilities were considered in the analysis. This shifting provision environment may also modify the expected relationship between its quality and contraceptive use.

The composite score accounts for facility-level structural factors affecting service quality. Including a woman’s visit from a CHW in the model allows for assessing public outreach services and accounts for an aspect of health system process quality measured at the individual level. CHW visits were significantly associated with use of modern and short-acting methods in Burkina Faso, Ethiopia and Uganda, but not in Kenya, and excluding the variable did not alter the quality score’s association with use (results not shown). This further reinforces the association identified between the quality score and contraceptive use, whereby the actual relationship between public service features and individual consumption is more nuanced than commonly perceived.

Several limitations of the analysis are to be noted. First, inferences about selected factors’ influence on the likelihood of contraceptive use are limited by the cross-sectional nature of the survey data. This design does not provide the needed temporality to establish causal effect of the quality of services on contraceptive behaviour. The study does, however, benefit from the absence of temporal ambiguity, present when facility and household surveys happen independently in time, given the identical period in which the facility and individual surveys were conducted by PMA2020. Secondly, generalizability of the findings is restricted to public facilities in Burkina Faso, Ethiopia, Kenya and Uganda and women for whom public facilities were the closest. The share of facilities that the sample of public SDPs represents differs by country and is more appropriate in Burkina Faso, Ethiopia and Uganda than in Kenya where the private sector is more developed. Thirdly, individually linking each woman to her closest facility strengthens the study’s ability to measure the effect of quality on individual contraceptive behaviour, but it requires the assumption that the closest service delivery environment influences contraceptive decisions. The study is limited by its inability to link each woman to the actual facility she is using. Finally, the measure of quality incorporates many structural and process variables available in the facility data set but does not capture provider and client characteristics that may influence the quality of services offered by facilities.

## Conclusion

This analysis has focused on assessing the role of the quality of family planning services on contraceptive practice, an aspect of fundamental importance for international initiatives that seek to increase contraceptive use. Specifically, it establishes a quantitative measure of quality by building a composite score for public facilities in Burkina Faso, Ethiopia, Kenya and Uganda. The index was also used to examine the effect of quality on contraceptive behaviour by linking data from the closest public facility to the woman’s data and a mixed relationship between quality and modern contraceptive use was found. Structural quality is positively and significantly associated with modern contraceptive use in Ethiopia and only positively but not significantly in Uganda. In Burkina Faso and Kenya, higher public facility service quality was associated with lower contraceptive use but only significant comparing high- to low-quality facilities in Kenya. However, process quality reflected in home visits by CHW, deployed from public facilities, was positively associated with modern contraceptive use in Burkina Faso, Ethiopia and Uganda. The patterns observed in this study may reflect the growing number of providers, including lower-level and informal providers, affecting the density of the service provision environment and perhaps modifying the relationship between facility-level quality and contraceptive use. Developing a quality score for private facilities through replication of the principal component analysis will be a beneficial next step in environments such as Kenya where the service provision environment cannot be summarized by the public sector. This analysis is illustrative of the first steps in creating a comprehensive methodology for the measurement of quality of family planning services in low-resource settings, such as in sub-Saharan Africa. Understanding the impact of family planning service quality on individual contraceptive behaviour continues to challenge the field while guiding programs to focus on it remains an important strategy to reducing unmet need for contraception.

## Ethical approval

As a secondary data analysis, this study was determined not to qualify as human subjects research and exempt from requiring approval by the Institutional Review Board of the Johns Hopkins Bloomberg School of Public Health (FWA #00000287).
